# A Facile Fabrication of ZnFe_2_O_4_/Sepiolite Composite with Excellent Photocatalytic Performance on the Removal of Tetracycline Hydrochloride

**DOI:** 10.3389/fchem.2021.736369

**Published:** 2021-08-16

**Authors:** Caihong Zhang, Xiaoyu Han, Fei Wang, Lijuan Wang, Jinsheng Liang

**Affiliations:** ^1^ Key Laboratory of Special Functional Materials for Ecological Environment and Information, Hebei University of Technology, Ministry of Education, Tianjin, China; ^2^ Institute of Power Source and Ecomaterials Science, Hebei University of Technology, Tianjin, China

**Keywords:** sepiolite nanofibers, ZnFe_2_O_4_, co-precipitation, photocatalytic, tetracycline hydrochloride

## Abstract

The excellent photo-response of ZnFe_2_O_4_ in the visible light region makes it a promising catalyst, whereas some defects like serious particle agglomeration and easy recombination of photo-generated electron-hole pairs hinder its application. In this work, the ZnFe_2_O_4_/sepiolite (ZF-Sep) composites were synthesized using a co-precipitation method. The obtained ZF-Sep composites were characterized by XRD, SEM, TEM, FT-IR, XPS, BET, VSM and DRS. Moreover, the photocatalytic performance was evaluated by the tetracycline hydrochloride removal efficiency under simulated visible light illumination. The results displayed that the ZnFe_2_O_4_ with average sizes about 20 nm were highly dispersed on sepiolite nanofibers. All the composites exhibited better photocatalytic performance than pure ZnFe_2_O_4_ due to the synergistic effect of the improvement on the agglomeration phenomenon of ZnFe_2_O_4_ and the reduction on the recombination rate of photo-generated electrons and holes. The optimum removal efficiency was that of the ZF-Sep-11 composite, which reached 93.6% within 3 h. Besides, the composite exhibited an excellent stability and reusability. Therefore, ZF-Sep composite is a promising catalyst for the treatment of wastewater contained antibiotics.

## Introduction

With the rapid expansion of pharmaceutical industry and breeding industry, the pollution of antibiotics and mycotoxins in the water environment has caused great concern. (Li et al., 2018; Das et al., 2020; Sun L. et al., 2020; Wang J. et al., 2020; Zhang et al., 2020). Because it is difficult to be metabolized by humans and animals, a large part of antibiotics are excreted in the form of urine and feces (Song et al., 2019). Hence, large quantities of antibiotics have been found in the soil, surface waters and even drinking water and will eventually threaten the health of humans and livestock through the food chain (Agerstrand et al., 2015; Isari et al., 2020a; Dong et al., 2020). Therefore, the removal of antibiotics from wastewater has been adopted, such as advanced oxidation processes (AOPs), membrane separation, microbial degradation, adsorption and photocatalysis (Debnath et al., 2020; Hayati et al., 2020; Khan et al., 2020; Wang Q. et al., 2020; Zhao R. et al., 2020; Zong et al., 2021). Among above techniques, photocatalytic degradation of antibiotics on the surface of photocatalytic has been received a lot of attention because of their simple operation, high efficiency, energy saving, environmental protection and mild reaction conditions (Isari et al., 2020b). Semiconductor-based photocatalysis has attracted much attention due to their effective photocatalytic performance and environmental friendliness (Li et al., 2017; Li et al., 2020.). Traditional semiconductor materials, such as TiO_2_, ZnO, ZrO_2_, Fe_2_O_3_ and CdS are the most commonly used photocatalytic materials due to their strong oxidizing ability, stable chemical properties, and high photocatalytic activity (He et al., 2020; Jain et al., 2020; Reddy et al., 2020; Hunge et al., 2021). However, the low adsorption capacity, easy recombination of photo-generated electron-hole pairs, insufficient visible-light absorption and difficulty of recycling of these semiconductor materials hinders their practical application (Sun Z. et al., 2020; Xiao et al., 2020).

Zinc ferrite (ZnFe_2_O_4_), a typical spinel material, which possesses an AB_2_O_4_ structure with Zn^2+^ ions occupy tetrahedral A and Fe^3+^ ions occupy octahedral B site in a face-centered cubic unit cell (Lima et al., 2018). Zinc ferrite is a promising semiconductor photocatalytic material due to its excellent photo-response in the visible light region, considerable chemical stability and easy recycling performance (Casbeer et al., 2012; Tsay et al., 2019; Borade et al., 2020; Hu et al., 2020). Mishra et al. used the co-precipitation method to synthesize spinel zinc ferrite (SZFO) atomic sheets. With the aid of microwave irradiation, it showed excellent degradation performance for bright green, and the degradation efficiency was greater than 99% within 5 min (Mishra et al., 2019). Sun et al. used micro-nano bubbles and recyclable MFe_2_O_4_ (where M = Mn, Zn, Cu, Ni and Co) synthesized by a hydrothermal method to simultaneously remove SO_2_ and NO_x_ from flue gas. The results show that MFe_2_O_4_ can accelerate the oxidation absorption of NO_x_ by producing OH. The NO_x_ removal efficiency increased from 32.85 to 83.88% in the NO_x_-SO_2_-MFe_2_O_4_-micro-nano bubble system, while the conversion rate of SO_2_ can reach 100% at room temperature (Sun and Li, 2020). Mesoporous zinc ferrite, agglomeration of nanoparticles with size of 5–10 nm, was prepared by Su et al. In the presence of visible light and hydrogen peroxide, the degradation efficiency of AOII reaches almost 100% within 2 h (Su et al., 2012). However, insufficient photo degradation activity was exhibited to use ZnFe_2_O_4_ merely under visible light (Han et al., 2019; Nguyen et al., 2019; Wu and Zhang, 2019). Nowadays, lots of works, such as ion doping and forming the composites with other substances, have been reported to improve the photo catalytic performance (Ajithkumar et al., 2019; Peymani-Motlagh et al., 2019; Zhao Y. et al., 2020). Different kinds of substances were used to combine with ZnFe_2_O_4_ to form composites, which mainly included metal oxides (ZnO, TiO_2_, Fe_2_O_3_, etc.) and carbon-based materials (reduced-graphene oxide, g-C_3_N_4_ and carbon nanotube, etc.). Moreover, the combination of ZnFe_2_O_4_ with more than one material has also attracted much attention. Enormous researches on the incorporation of ZnFe_2_O_4_ with mental oxides and carbon materials, silver species and carbon materials and inorganic-organic composite materials have been published recently. The introduction of these substances greatly improves the physical and chemical structure of ZnFe_2_O_4_, so that the optical, magnetic, catalytic and other properties were optimized (Adnan et al., 2021; Feng et al., 2021; Sun et al., 2017; Zia et al., 2020; Baynosa et al., 2020; Wang and Nan, 2019; Qin et al., 2017.). Compared with above mentioned substances, natural mineral materials have many advantages such as large specific surface area, abundant pore structure, strong adsorption capacity, large abundance and low cost, thereby they have great potential as catalyst carrier materials (Hu et al., 2019; Zhou et al., 2020).

Sepiolite, as a hydrous magnesium-rich silicate [Si_12_Mg_8_O_30_(OH)_4_(OH2)_4_⋅8H_2_O], is a typical fibrous natural clay mineral with a layered chain structure. Sepiolite is composed of two silicon-oxygen tetrahedrons sandwiching a magnesium-oxygen octahedron and the discontinuity of the silicon-oxygen tetrahedron makes the sepiolite have a rich internal tunnel structure. Benefit from the unique structure and composition, sepiolite fibers possess large specific surface area, high porosity and various functional groups, which provides more reaction sites for supported catalysts (Ma and Zhang, 2016; Wang et al., 2017; Zhang et al., 2017; Mishra et al., 2019; Cui et al., 2020). In addition to its abundant storage, low cost and environmental friendliness, sepiolite is an ideal candidate for catalyst carrier (Xu et al., 2017). However, in order to increase surface area and enlarge its pore structure as well as to increase the number of active sites, an acid treatment is considered necessary before the synthesis process on raw sepiolite material. Therefore, the acid-treated sepiolite was often used to the support material for metal oxides TiO_2_, ZnO, Fe_3_O_4_, Cu_2_O, etc. (Xu et al., 2010; Zhu et al., 2012; Daneshkhah et al., 2017; Xu et al., 2019; Wang S. et al., 2020). In our pervious study, we have prepared CoAl_2_O_4_/sepiolite nanofibers composite and Co./CoAl_2_O_4_/sepiolite nanocomposite *via* different methods (Zhang et al., 2018; Wang F. et al., 2019; Hao et al., 2021; Wang et al., 2021). Nevertheless, as far as we know, there were few reports on sepiolite loaded ZnFe_2_O_4_ as a catalyst.

In this work, the ZnFe_2_O_4_ nanoparticles were grown on sepiolite nanofibers to obtain ZnFe_2_O_4_/sepiolite (ZF-Sep) composites by a co-precipitation method. Through different characterization of the composite and its photocatalytic performance for TCH, the influence of the structure of the composite on its performance was explored. This study provides a new idea for improving the performance of the catalysts and an inspiring approach for cost-effective preparation of highly efficient catalysts for wastewater containing antibiotics.

## Expreimental Section

### Materials

Raw sepiolite was purchased from Henan province, and the main chemical compositions analyzed by XRF were 54.36% SiO_2_, 35.6% MgO, 5.67% CaO and 1.36% Fe_2_O_3_. Hydrochloric acid (HCl), iron (III) nitrate nonahydrate [Fe(NO_3_)_3_⋅9H_2_O], zinc chloride (ZnCl_2_), ammonia (NH_3_⋅H_2_O), ethanol (CH_3_CH_2_OH), silver nitrate standard solution (AgNO_3_, 0.1 mol/L), tetracycline hydrochloride (TCH), butyl alcohol (TBA), P-benzoquinone (BQ) and ammonium oxalate (AO) were analytical reagent and used without further purification.

### Synthesis of ZnFe_2_O_4_ and ZF-Sep Composites

**FIGURE 1:**
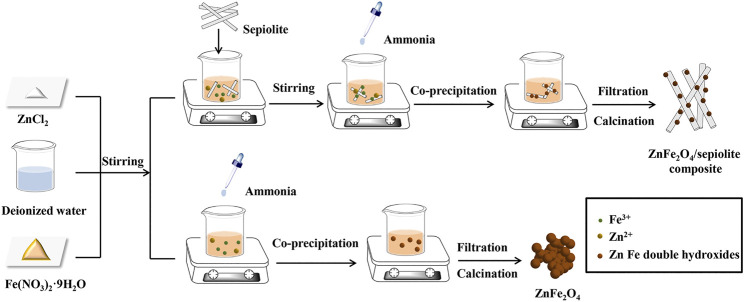
Schematic for the fabrication of ZF-Sep composite.

1.3629 g of ZnCl_2_ and 8.08 g of Fe(NO_3_)_3_⋅9H_2_O were dissolved in 100 ml deionized water, and a certain amount of sepiolite which were prepared by an acid treatment were added into the solution. Next, the pH of suspension was adjusted to 11 by adding aqueous ammonia dropwise. After aging for 12 h at room temperature, the precursor slurry was washed with ethanol and deionized water until the presence of chloride ions cannot be detected with silver nitrate standard solution. Then the filter cake was calcined in a muffle furnace for 3 h at 600°C. Finally, the ZnFe_2_O_4_/sepiolite composite (ZF-Sep) was obtained, and the schematic diagram was shown in Figure 1. On the other hand, pure ZnFe_2_O_4_ were also prepared by the same process. Samples were prepared with initial mass ratios of ZnFe_2_O_4_ to sepiolite nanofibers having values of 1:3, 1:2, 1:1, 2:1, 3:1, and labeled as ZF-Sep-13, ZF-Sep-12, ZF-Sep-11, ZF-Sep-21, ZF-Sep-31, respectively.

### Characterization

Element analysis was carried out by ZSX Primusll X-ray fluorescence spectrometer (XRF). X-ray diffraction (XRD) patterns were employed to analyze the phase composition of samples by an X’Pert MPD dilatometer with CuKα radiation (40 Kv, 40 mA and *λ* = 1.54180 Å). The scanning was made in the 2θ range of 5–90° with a scanning speed of 12/min at room temperature. Scanning electron microscopy (SEM, JSM 7610F) and transmission electron microscopy (TEM, JEM-2010FEF, JEOL) were employed to observe the morphologies of the samples. Infrared radiation spectra of the as-prepared composites were obtained by a Fourier transform-infrared (FTIR) test spectrometer (Bruker VERTEX 80V) in the range of 4,000–400 cm^−1^ using KBr pellets. The X-ray photoelectron spectroscopy (XPS) measurements were performed on ESCALAB 250Xi (United States, Thermo Fisher Scientific) using a monochrome Al Kα (150 W, 20 eV pass energy, and 500 μm beam spot size). The magnetic property of the samples was measured by a vibrating sample magnetometer (VSM, Lakeshore VSM 7407) at room temperature. The surface area of the samples was tested by the physicochemical adsorption analyzer (United States, autosorb iQ). Diffuse reflectance ultraviolet-visible spectra (UV-vis DRS) were measured on a Shimadzu UV-1800 spectrophotometer.

### Photocatalytic Performance

The photocatalytic performance of the as-prepared samples was evaluated by the removal efficiency of TCH under visible light irradiation. 0.1 g of catalyst was dispersed into 100 ml of TCH solution (20 mg/L). Before the suspension was subjected to irradiation by a 300 W Xe lamp (*λ* > 420 nm), stirring the produced suspension in the dark for half an hour to reach the adsorption/desorption equilibrium. Then, 3 ml of the suspension were extracted every 30 min and passed through a 0.22 micron filter membrane to remove the catalysts. The absorbance values at 357 nm of the filtrate were measured by a TU-1800 ultraviolet visible spectrophotometer. The removal efficiency can be calculated according to the following equation:

$$RE\%=\frac{\left(C_{0}-C_{t}\right)}{C_{0}}\times 100\text{\% }$$ (1)

Where RE% represents the removal efficiency of catalyst, *C*_*0*_ represents the TCH concentration at the beginning, and *C*_*t*_ represents the TCH concentration at a certain time *t*.

To detect the active species generated in the degradation process, the scavengers including butyl alcohol (TBA; 5 mmol/L), *p*-benzoquinone (BQ; 5 mmol/L), and ammonium oxalate (AO; 5 mmol/L) were added into the solution of TCH, respectively. The photocatalytic process was the same as that described above.

## Results and Discussion

### Characterization of ZnFe_2_O_4_ and ZnFe_2_O_4_/Sepiolite Composites

**FIGURE 2:**
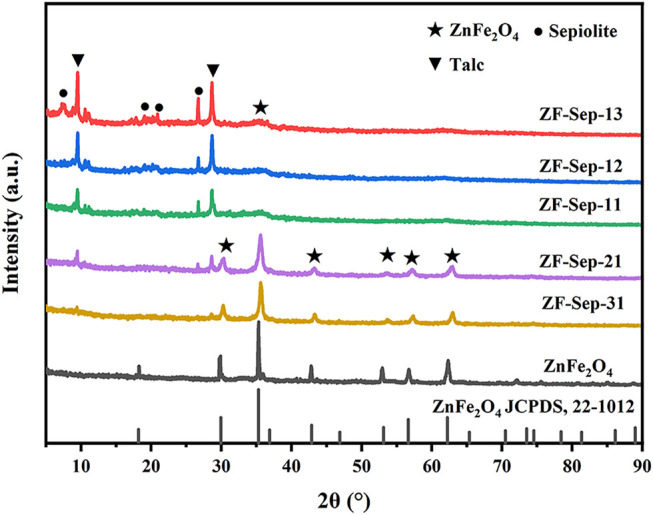
XRD patterns of ZnFe_2_O_4_ and ZF-Sep composites with different ZnFe_2_O_4_ loadings.

The phase composition of samples was examined by XRD analysis. As shown in Figure 2, seven diffraction peaks (2θ) at 18.25°, 29.66°, 35.30°, 42.83°, 52.94°, 56.71° and 62.35° in curve of ZnFe_2_O_4_ correspond to the planes (111), (220), (311), (400), (422) (511) and (440) of ZnFe_2_O_4_, respectively. It confirms that single phase ZnFe_2_O_4_ (JCPDS No. 22-1012) with cubic spinel structure were synthesized successfully (Ma et al., 2019; Madhukara Naik et al., 2019). The characteristic peaks of sepiolite gradually decreased with the increase of the ZnFe_2_O_4_ content in the ZF-Sep composites, whereas the peaks intensity of ZnFe_2_O_4_ strengthened gradually, indicating that the co-existence of ZnFe_2_O_4_ and sepiolite in these composites. The decrease and broadening of diffraction peaks of ZnFe_2_O_4_ was derived from its dispersing in the surface of sepiolite. The decrease of sepiolite peak intensity was attributed to its imperfect crystalloid by disconnecting the fiber unit and the phase change of sepiolite to talc at the sintering temperature of 600°C (Xu et al., 2010).

**FIGURE 3:**
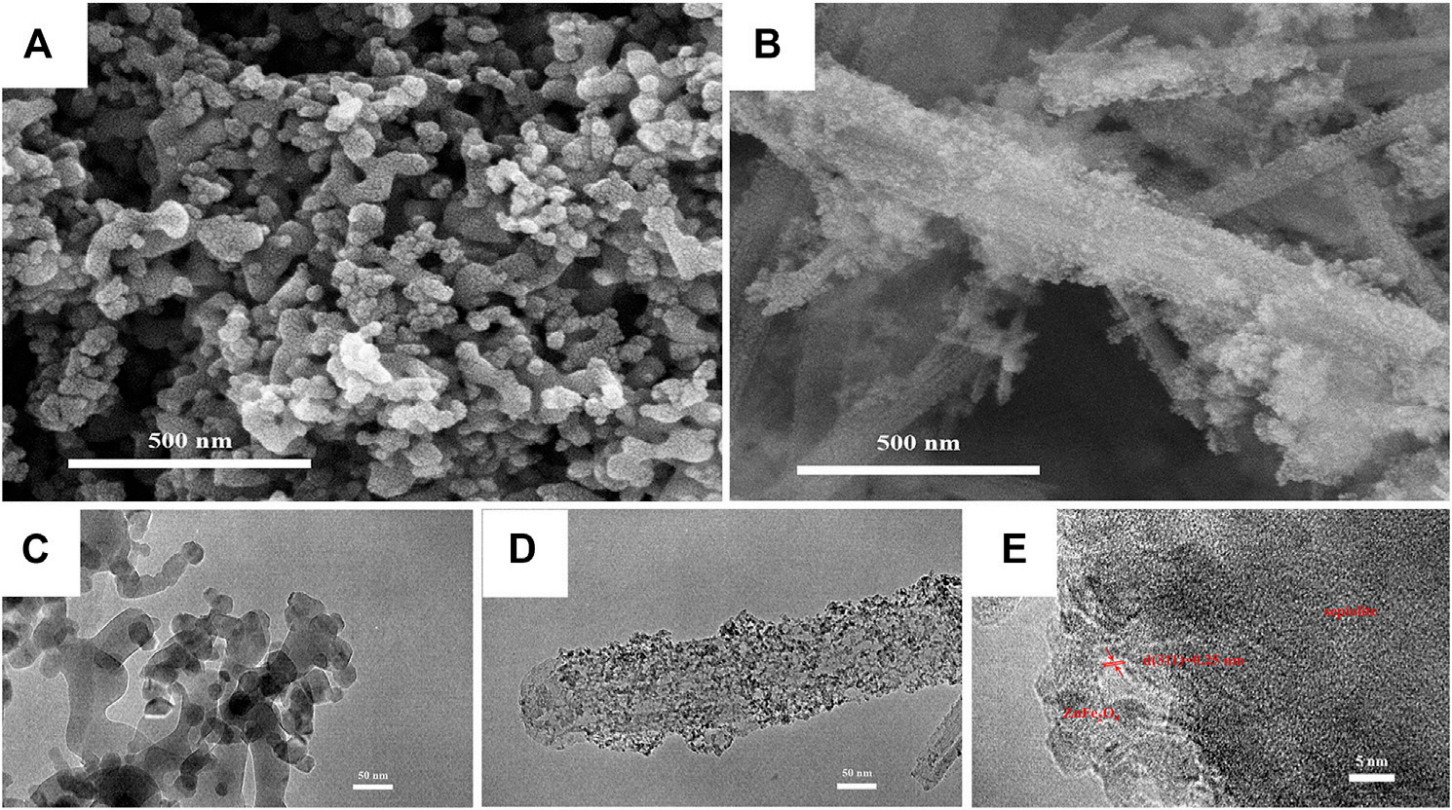
SEM images of **(A)** ZnFe_2_O_4_ **(B)** ZF-Sep-11; TEM images of **(C)** ZnFe_2_O_4_ **(D)** ZF-Sep-11; HRTEM images of **(E)** ZF-Sep-11.

**FIGURE 4:**
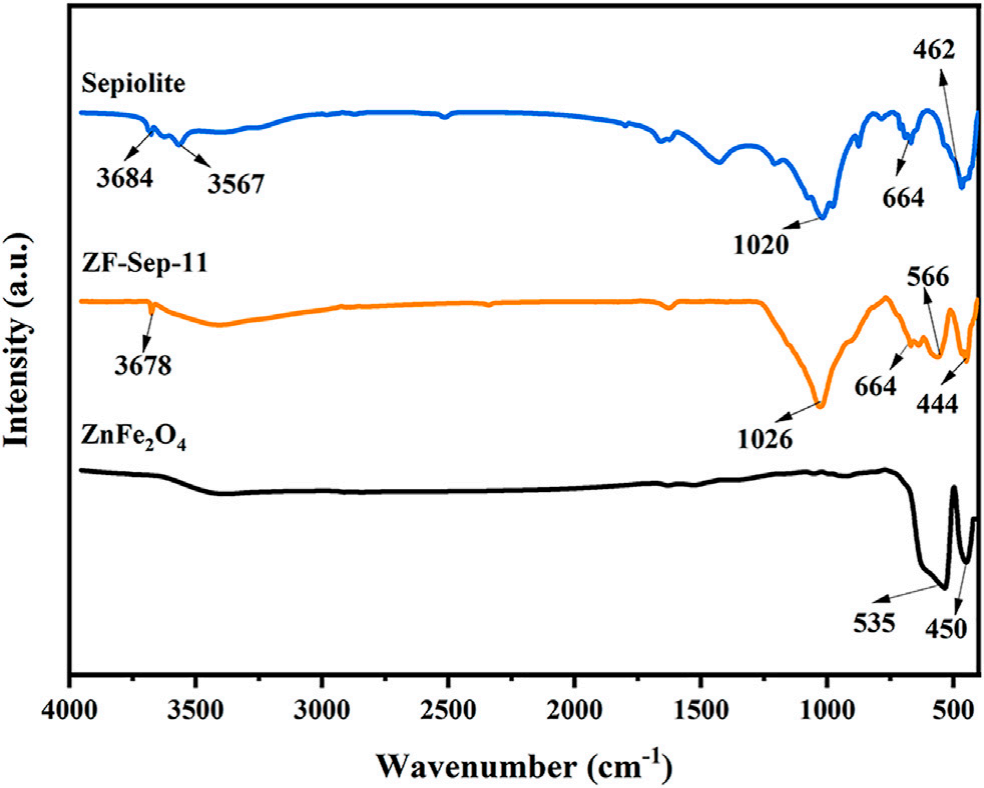
FT-IR spectra of ZnFe_2_O_4_, Sepiolite and ZF-Sep-11.

The micromorphology of the ZnFe_2_O_4_ and ZF-Sep-11 composite were characterized by SEM and TEM. In Figures 3A,C, the images show that ZnFe_2_O_4_ sample was consisted of irregular nanoparticles with a size of about 20–200 nm. The existence of relatively large particles was attributed to the high specific surface energy of the nanoparticles causing serious agglomeration. As seen in Figures 3B,D, lots of small and irregular particles (about 20 nm) attached to the surface of sepiolite fibers, and the high resolution image (Figure 3E) displayed that the interface of ZnFe_2_O_4_ possessing obvious lattice fringes [d (311) = 0.25 nm] closely connected with the interface of sepiolite which shows no obvious lattice fringes due to the low crystallinity. Consistent with the XRD results, SEM and TEM analysis also confirmed the successful synthesis of the composite, and the introduction of sepiolite fibers largely alleviated the agglomeration of ZnFe_2_O_4_. Figure 4 shows the FTIR spectra of the sepiolite, ZnFe_2_O_4_ and ZF-Sep-11 composite. In the spectra of sepiolite, the bonds at 3,684–3,567 cm^−1^ and 664 cm^−1^ corresponded to the stretching and bending vibrations of Mg-OH in the Mg-O octahedral sheet. The bonds at 1,020 and 462 cm^−1^ were attributed to the stretching vibrations of Si-O bond in the Si-O-Si groups of the Si-O tetrahedral sheet (Zhang et al., 2014; Zhu et al., 2012.). There were two obvious peaks at 535 and 450 cm^−1^ in the spectra of ZnFe_2_O_4_, which could be ascribed to the stretching vibrations of the Zn-O bond and Fe-O bond in the spinel structure (Li J. et al., 2019; Mohan et al., 2020; Wang P. et al., 2019.). As shown in the spectra of ZF-Sep-11, the stretching vibrations of Si-O bond at 1,020 cm^−1^ shifted to 1,026 cm^−1^ and the stretching vibrations of Zn-O and Fe-O bands at 535 and 450 cm^−1^ shifted to 566 and 444 cm^−1^, which was possible ascribed to the interaction between ZnFe_2_O_4_ and sepiolite nanofibers (Wang W. et al., 2019; Xu et al., 2017.).

**FIGURE 5:**
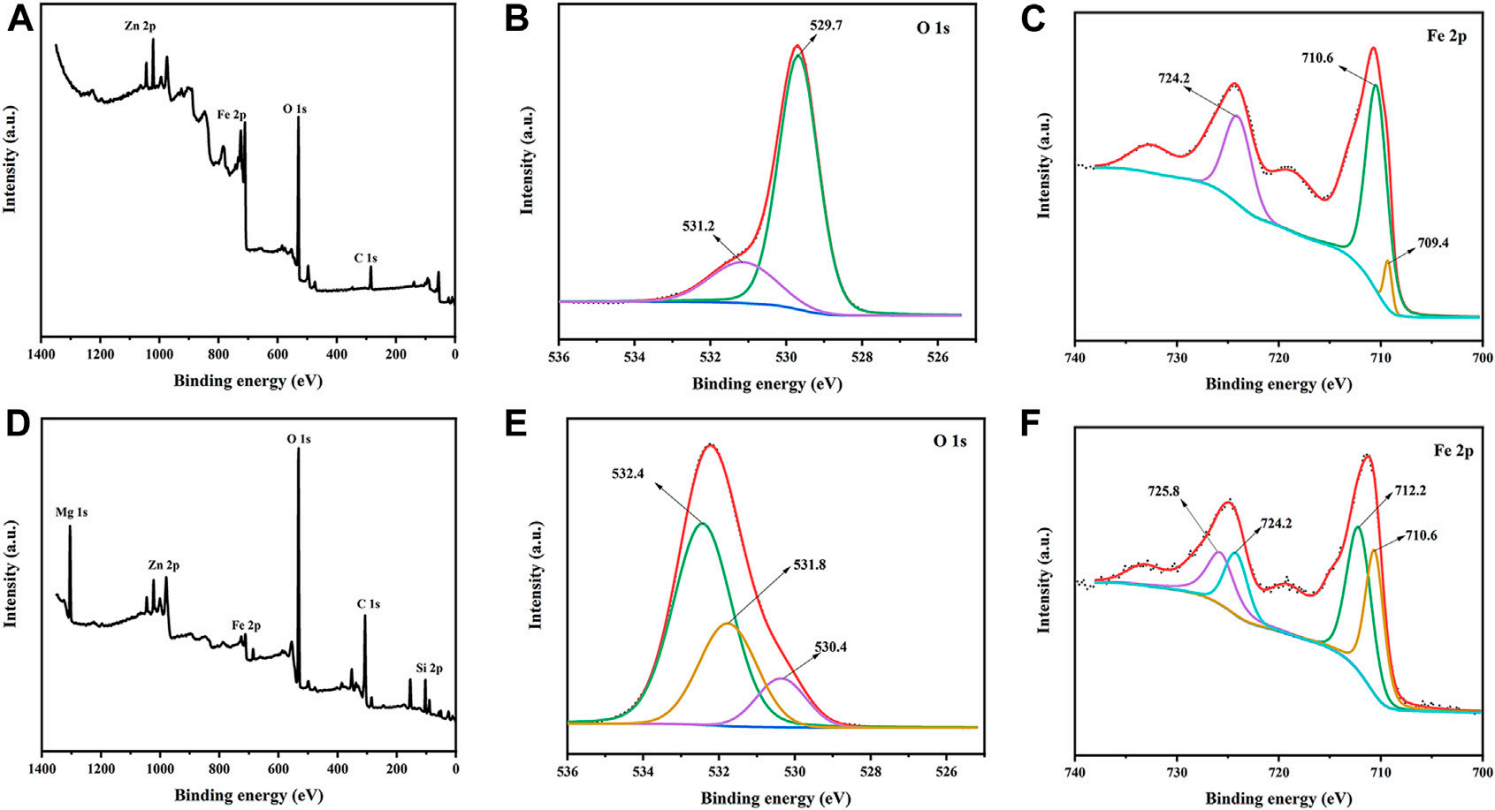
XPS spectra of ZnFe_2_O_4_ **(A)** survey **(B)** O 1s spectrum **(C)** Fe 2p spectrum; and ZF-Sep-11 **(D)** survey **(E)** O 1s spectrum **(F)** Fe 2p spectrum.

The XPS method was employed to the ZnFe_2_O_4_ and ZF-Sep-11 composite sample to investigate its elemental composition and chemical states. As illustrated in Figure 5A, the survey spectrum of ZnFe_2_O_4_ shows the signal peaks of Fe 2p, Zn 2p, O 1s, indicating that Zn, Fe and O elements in the samples. Compared with ZnFe_2_O_4_, the appearance of Mg 1s and Si 2p indicated the introduction of sepiolite (Figure 5D). It is worth noting that the signal peaks of C 1s in the XPS survey spectrum are mainly caused by the external C impurities of XPS instrument. In Figure 5B, the O 1s spectrum of ZnFe_2_O_4_ could be divided into two peaks at approximately 529.7 and 531.2 eV, corresponding to the lattice oxygen and the oxygen absorbed on the surface, respectively (Wang S. et al., 2020). As shown in Figure 5E, the peak at 530.3 eV represented the lattice oxygen of ZnFe_2_O_4_, and that at 531.8 and 532.4 were attributed to the O atom of the -OH and Si-O-Si bond from sepiolite nanofibers. In the Fe 2p spectrum of ZnFe_2_O_4_ (Figure 5C), the peaks at 724.2 and 710.6 eV represented the Fe^3+^, and 709.4 eV were attributable to the Fe^2+^ (Li Y. et al., 2019; Wang S. et al., 2020). Compared with ZnFe_2_O_4_, the peak positions of Fe^3+^ (725.8 and 712.2 eV) and Fe^2+^ (724.2 and 710.6 eV) had a certain shift, and the ratio of Fe^3+^ to Fe^2+^ was reduced (Figure 5F), which could be ascribed to the electron transfer and ion exchange between ZnFe_2_O_4_ and sepiolite nanofibers. Therefore, the sepiolite nanofibers in the composite might act as a good medium for the migration of photogenerated carriers in the reaction process, thereby reducing the recombination rate of photogenerated electrons and holes to increase the photocatalytic efficiency (Liu et al., 2015).

**FIGURE 6:**
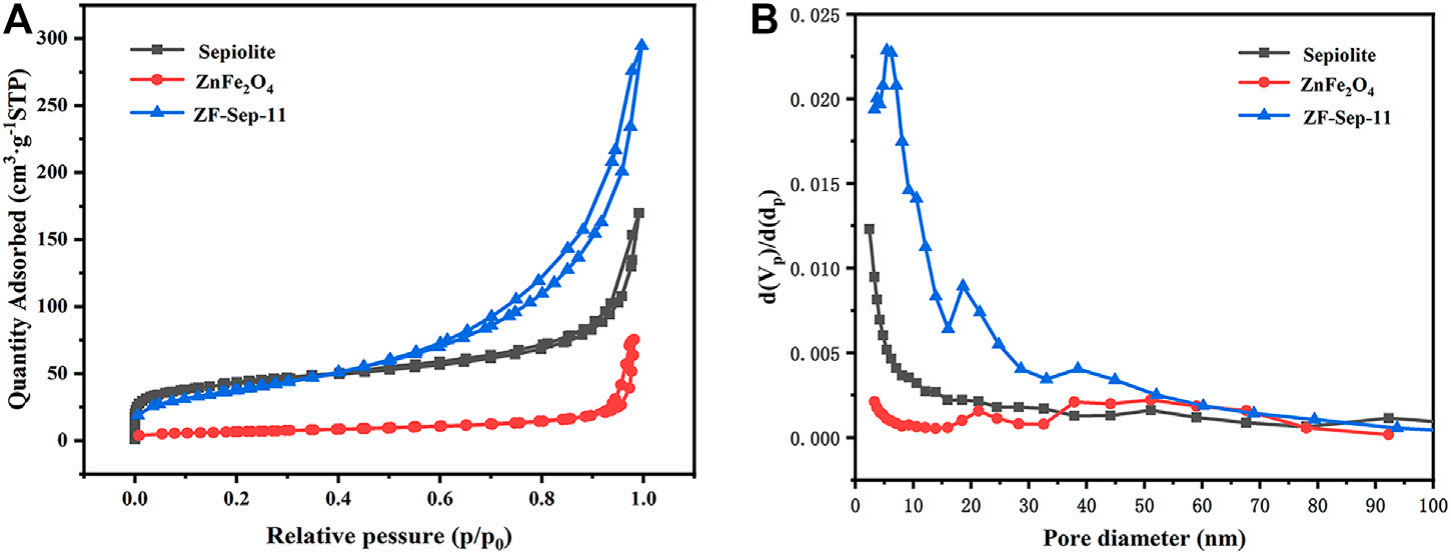
**(A)** The N_2_ adsorption-desorption isotherms, and **(B)** pore size distribution of sepiolite, ZnFe_2_O_4_ and ZF-Sep-11.

The nitrogen adsorption-desorption isotherms of sepiolite, ZnFe_2_O_4_ and ZF-Sep-11 composite were shown in Figure 6A, and the isotherms were in the shape of type IV, which indicated a typical of mesoporous materials. The result was further confirmed by the corresponding pore size distribution in Figure 6B. The specific surface area, total pore volume and average pore size of ZnFe_2_O_4_ and ZF-Sep composites with different loadings of ZnFe_2_O_4_ were summarized in Table 1. With the increase of the ZnFe_2_O_4_ loadings, the specific surface area of the composites showed a trend of first increasing and then decreasing, but all the composites were larger than pure ZnFe_2_O_4_. The optimal sample was ZF-Sep-11 composite, and its specific surface area was 138.3 m^2^g^−1^. The nitrogen adsorption-desorption isotherms also demonstrated that the introduction of sepiolite nanofibers improved the agglomeration of ZnFe_2_O_4_, which increased the contact area with the target degradation product, thereby improved the photocatalytic performance.

**TABLE 1:** Textural parameters of ZnFe_2_O_4_, and the ZF-Sep composites with different contents of ZnFe_2_O_4_.

Sample	Specific surface area (m^2^g^−1^)	Total pore volume (cm^3^g^−1^)	Average pore diameter (nm)
ZnFe_2_O_4_	23.5	0.12	51.1
ZF-Sep-13	95.9	0.37	3.7
ZF-Sep-12	117.5	0.37	3.7
ZF-Sep-11	138.3	0.43	5.4
ZF-Sep-21	75.6	0.36	10.5
ZF-Sep-31	70.0	0.34	13.9

**FIGURE 7:**
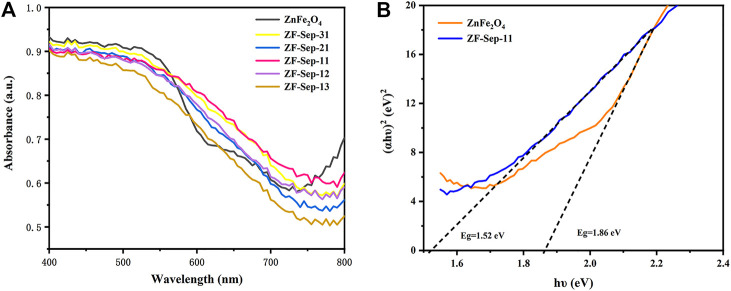
**(A)** UV-vis spectra of ZnFe_2_O_4_ and ZF-Sep composites **(B)** Plots of (αhν) ^2^ vs hν of ZnFe_2_O_4_ and ZF-Sep-11.

The optical properties of the as-prepared samples were analyzed by UV-vis reflectance spectroscopy to evaluate the light absorption ability. As shown in Figure 7A, ZnFe_2_O_4_ and ZF-Sep composites presented significant absorbance in the 450–700 nm wavelength range. The band gap of ZF-Sep-11 composite could be estimated to be 1.52 eV, which was a little smaller than that of ZnFe_2_O_4_ (1.86 eV) (Figure 7B). In comparison with ZnFe_2_O_4_, ZF-Sep-11 composite showed the narrower band gap and higher light absorption, which could exhibit positive influence on the removal efficiency of target antibiotic in the visible light range.

**FIGURE 8:**
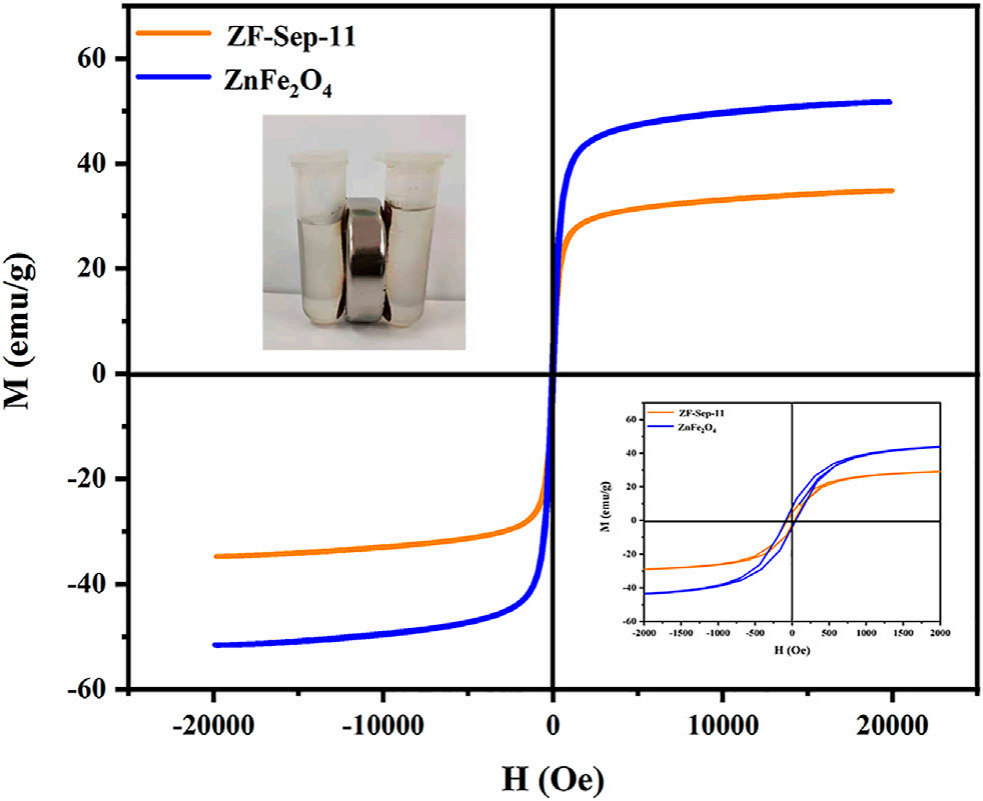
Magnetization curves of ZnFe_2_O_4_ and ZF-Sep-11, inset show zoomed in version and the situation with an external magnetic field.

The magnetic properties of the ZnFe_2_O_4_ and ZF-Sep-11 composite were measured by a vibrating sample magnetometer (VSM) with an applied magnetic field between −20,000 and 20,000 Oe at room temperature. Figure 8 shows the plot of magnetization *versus* applied field with a small hysteresis loop which indicates that the samples display typical ferromagnetic (soft magnetic). The saturation magnetization (M_s_) of ZnFe_2_O_4_ and ZF-Sep-11 composite were 51.693 and 34.780 emu/g, respectively. The decreasing of M_s_ mainly derives from the addition of non-magnetic material sepiolite. Due to the typical ferromagnetic, the catalyst could be efficiently removed from the aqueous solution of reaction mixture by an external magnet.

### Photocatalytic Performance

**FIGURE 9:**
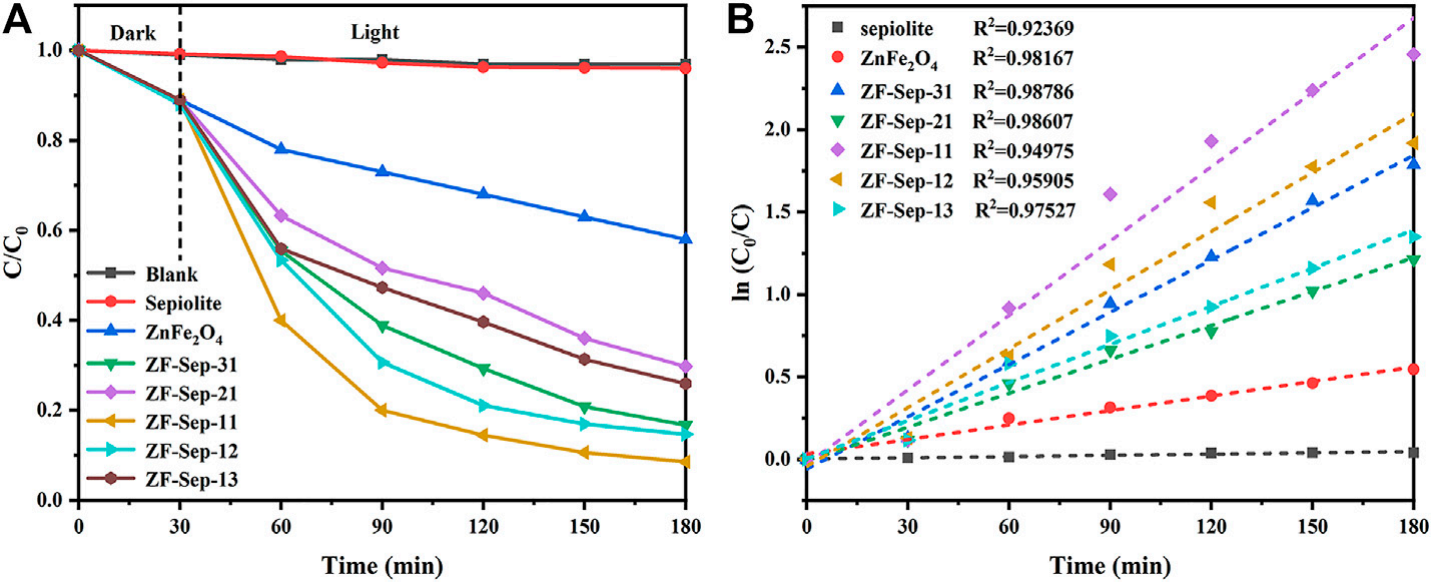
**(A)** Removal of TCH under different systems, and **(B)** ln (C_0_/C) *versus* reaction time for TCH under different conditions. Reaction conditions (TCH) = 20 mg/L (catalysts) = 1.0 g/L (H_2_O_2_) = 1 mM.

The photocatalytic performance of the as-synthesized samples was evaluated by degrading TCH under visible light (*λ* ≥ 420 nm). The measured removal efficiency of the samples under different preparation conditions were depicted in Figure 9. As can be seen in Figure 9A, the removal efficiency of TCH solution was almost negligible when there was no catalyst and single sepiolite. For ZnFe_2_O_4_ and the composites, the decrease in the concentration of the TCH solution in the first 30 min in the absence of light may be due to the influence of the Fenton system formed by the addition of H_2_O_2_. The removal efficiency of tetracycline hydrochloride was 56.7% for ZnFe_2_O_4_ within 3 h. All of the composites exhibited superior removal efficiency for TCH compared with the single ZnFe_2_O_4_. Among these composites, ZF-Sep-11 showed the optical performance, and the removal efficiency of TCH reached 93.6% within 3 h. Figure 9B shows the reaction kinetics of the as-synthesized samples, in which the experimental data were in accordance with the pseudo first-order kinetic equation:

$$\text{ln}\left({\text{C}}_{0}/\text{C}\right)=\text{kt}$$ (3)

Where *C*_*0*_ is initial concentration of TCH solution, *C* is the concentration of tetracycline hydrochloride at reaction time *t*, and *t* is the reaction time and *k* is the fitted kinetic rate constant. The values of kinetic rate constant of sepiolite, ZnFe_2_O_4_, ZF-Sep-31, ZF-Sep-21, ZF-Sep-11, ZF-Sep-12 and ZF-Sep-13 were 0.000248, 0.00293, 0.01057, 0.00686, 0.01504, 0.01188 and 0.00771 min^−1^, respectively. ZF-Sep-11 showed the highest kinetic rate constant, which is about five times higher than that of signal ZnFe_2_O_4_.

### Influence of Reaction Factors on Removal Efficiency of TCH

**FIGURE 10:**
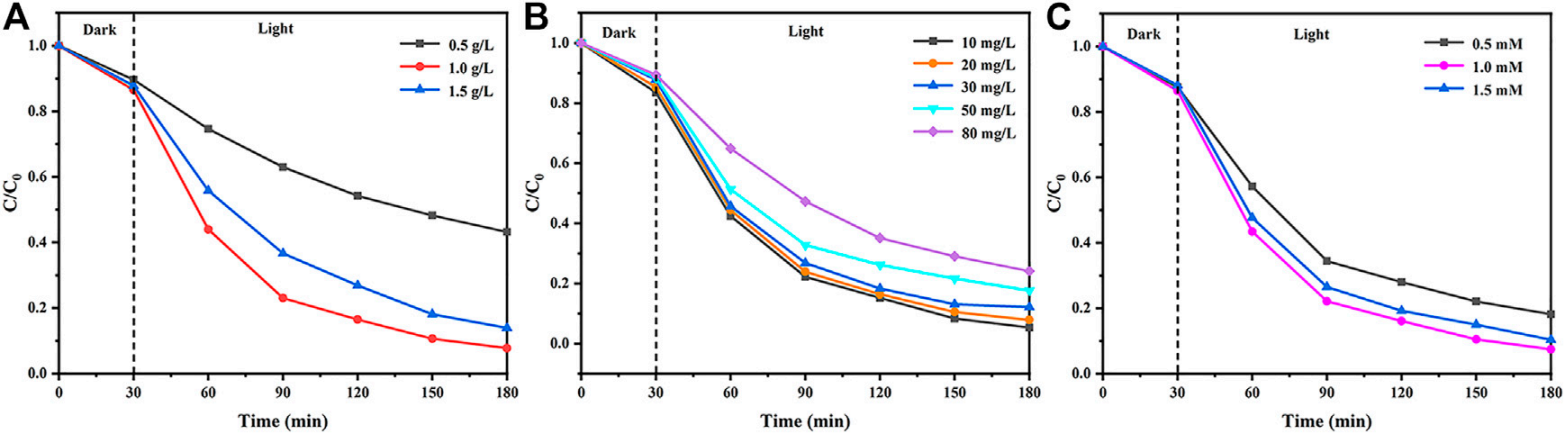
Effect of **(A)** catalyst dosage **(B)** initial concentration, and **(C)** amount of H_2_O_2_ on the removal of TCH for ZF-Sep-11. Reaction conditions (TCH) = 20 mg/L (catalysts) = 1.0 g/L (H_2_O_2_) = 1 mM.

Different amounts of ZF-Sep-11 composite were used in the catalytic experiment to explore the effect of the catalyst content on the removal efficiency of TCH. The dosage of catalyst is set to 0.5, 1.0 and 1.5 g/L (the ratio of catalyst to TCH solution). In Figure 10A, when the dosage of catalyst was 0.5 and 1.5 g/L, the removal efficiency was 66.8 and 86.2% at 3 h, respectively, which were lower than the removal efficiency of 1.0 g/L (92.3%). The result means that too little catalyst dosage will cause the reduction of removal efficiency, because a small amount of active free radicals were generated. However, when an excessive amount of the catalyst was dispersed in the TCH solution, a small amount of light can reach their surface due to the influence of turbidity and scattering effect. The less exposed area under light may result in a decrease in overall catalytic efficiency.

Figure 10B showed the removal efficiency at different initial concentrations of TCH solution. The removal efficiency was 94.7, 92.2, 87.9, 82.4 and 75.9% in 3 h for 10, 20, 30, 50 and 80 mg/L of TCH solution, respectively. As the concentration increases, the removal efficiency of TCH gradually decreases. It could be attributed to the fact that the active sites on the surface of the catalyst are blocked in a high-concentration tetracycline solution.

In order to improve the ability to remove TCH, the amount of H_2_O_2_ added has been optimized. In Figure 10C, compared with the addition of 1 mM, when the addition of H_2_O_2_ was 0.5 and 1.5 mM, the removal efficiency were slightly reduced. Low H_2_O_2_ addition produces little free radicals. However, the excess H_2_O_2_ molecules will act as a quencher of OH to generate perhydroxyl (·OOH) radicals and compete with OOH to generate H_2_O and O_2_ (Su et al., 2012).

$$\cdot \text{OH }+{\text{H}}_{2}{\text{O}}_{2}\to {\text{H}}_{2}\text{O}+\cdot \text{OOH}$$ (4)

$$\cdot \text{OH }+\cdot \text{OOH}\to {\text{H}}_{2}\text{O}+{\text{O}}_{2}$$ (5)

### Reactive Species of TCH Removal

**FIGURE 11:**
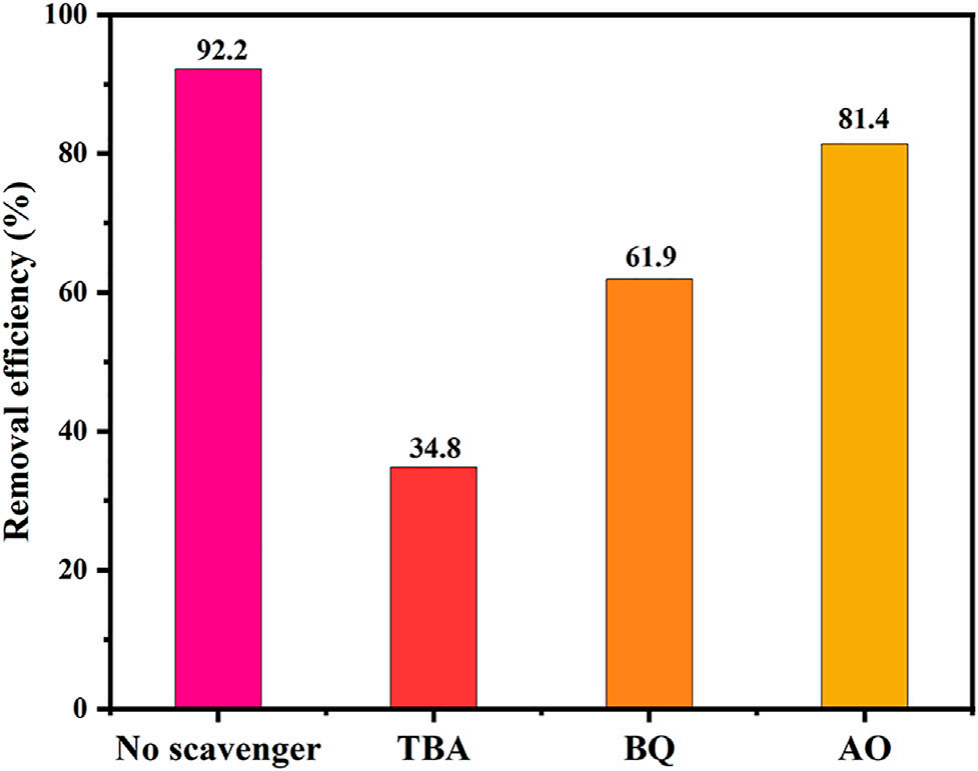
Active species trapping experiment of ZF-Sep-11. Reaction conditions (TCH) = 20 mg/L [catalysts] = 1.0 g/L (H_2_O_2_) = 1 mM (TBA) = 5 mmM (BQ) = 5 mmM (AO) = 5 mmM.

In order to determine the main active species in the removal of TCH for ZF-Sep composite, free radical trapping experiments were implemented. BQ, IPA and AO were added as scavengers for O_2_^−^, ·OH and h^+^, respectively. As depicted in Figure 11, the removal efficiency of TCH was 93.2% without any scavengers. After adding AO, there was no obviously decline in the removal efficiency of TCH (81.4%). However, the addition of TBA and BQ decreased the removal efficiency of TCH to 34.8 and 61.9%, respectively. The above results indicate that O_2_^−^ and OH were the main active species in the removal process.

### Possible Degradation Pathway

**FIGURE 12:**
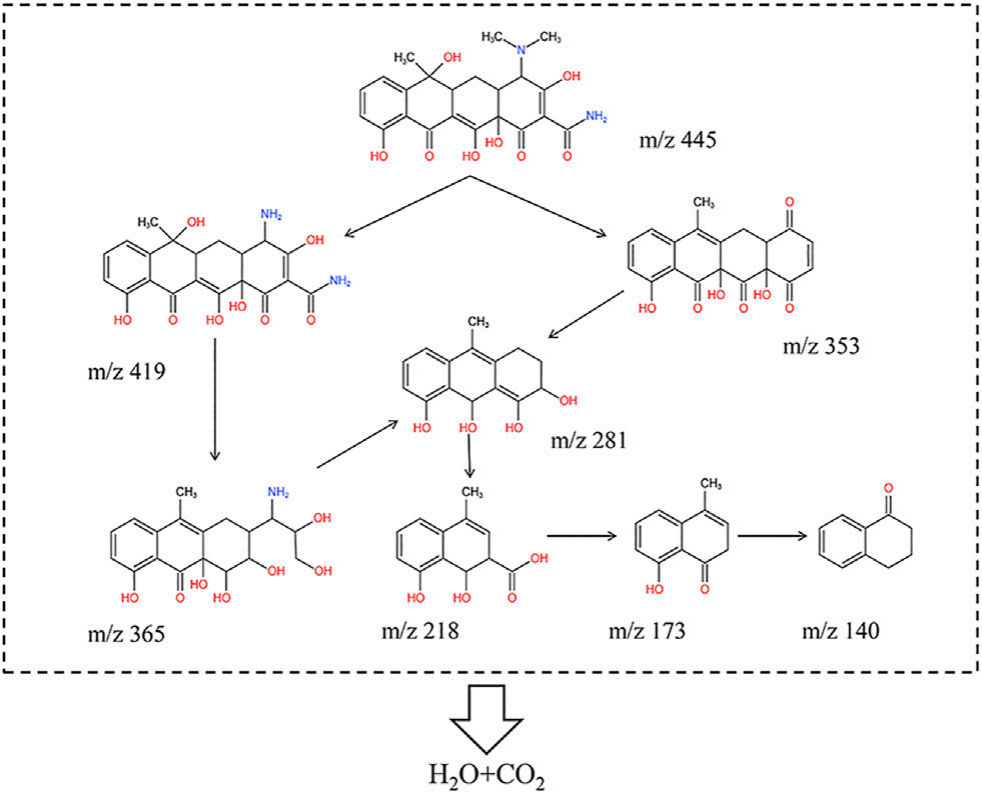
Intermediates and reaction pathways of TCH photodegradation under visible light illumination over ZF-Sep-11.

The liquid chromatography-mass spectroscopy (LC-MS) was used to analyze the possible intermediates that produced during the TCH degradation process to reveal the possible TCH degradation pathway. The LC-MS spectra displayed the formation of intermediate products with m/z values of 445, 419, 365, 353, 279, 218, 173, and 140 under visible light irradiation. According to the above results, the possible TCH degradation paths were proposed as shown in Figure 12. Firstly, TCH dissociates into tetracycline (TC) corresponding to m/z 445 in the aqueous solution (Lu et al., 2021). Due to the produced active species easily attack the amine group, hydroxyl group and methyl group in TC, the mass spectra corresponding to m/z 419 and m/z 353 were identified as the products formed from detachment of these groups of TC molecule. Secondly, the ring-opening products (m/z 365, m/z 281 and m/z 218) were assigned as the further oxidation products. Carboxyl group was detached from the ring-opening product (m/z 270) and then the intermediate product (m/z 140) was formed from the demethylation reaction (Pang et al., 2018; Li Z. et al., 2019.). Finally, these intermediate products were mineralized into CO_2_ and H_2_O *via* ring-opening reactions (Wu et al., 2021.).

### Possible Mechanism

**FIGURE 13:**
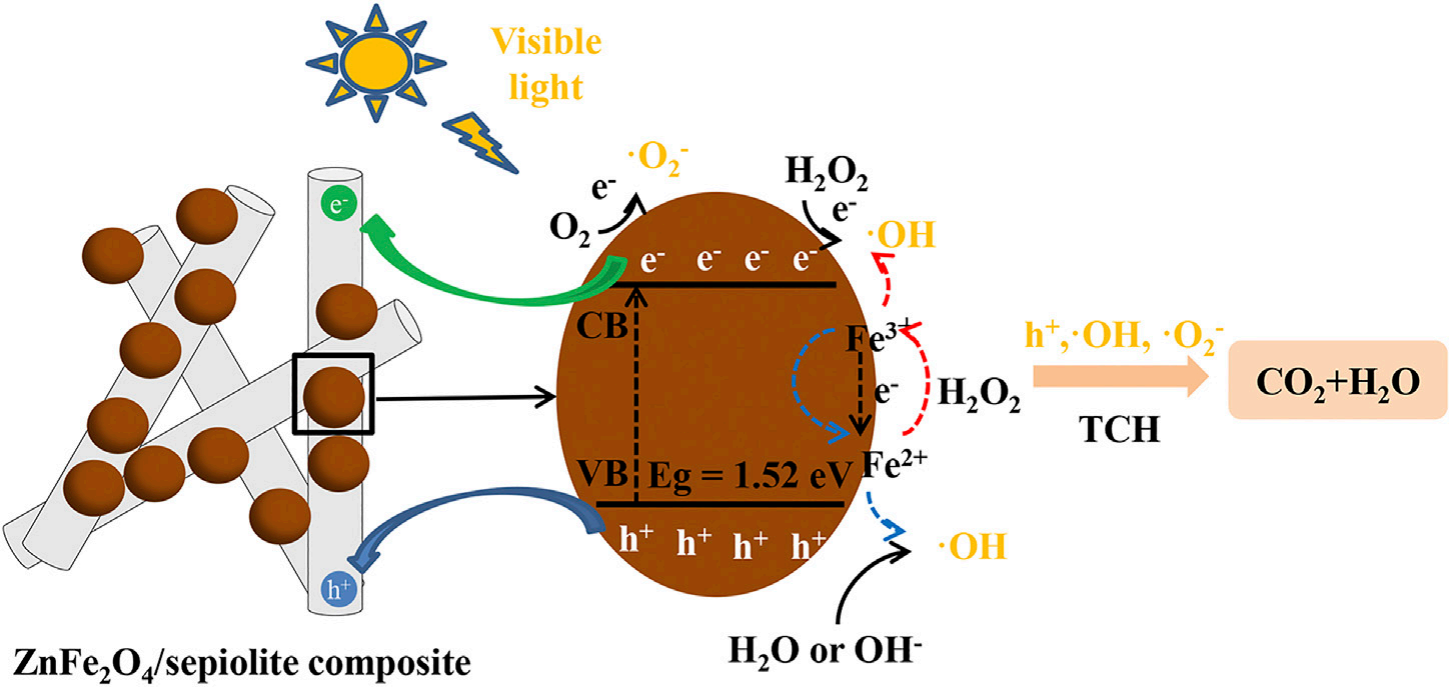
Schematic diagram of possible mechanism.

On the basis of the above analysis, a possible mechanism was shown in Figure 13. The loading of ZnFe_2_O_4_ on sepiolite nanofibers significantly improves its agglomeration phenomenon, which made more active sites in the surface of ZnFe_2_O_4_ were exposed, thereby improving its catalytic activity. Under visible light, the catalyst was activated to generate electron-hole pairs (Eq. 6). The sepiolite nanofibers might act as a good medium for the migration of photogenerated carriers to reduce the recombination rate of photogenerated electrons and holes. The holes were captured by OH^−^ or H_2_O to generate OH, and O_2_^−^ radicals were generated by trapping electrons for O_2_ (Eqs. 7–9). In the presence of H_2_O_2_, it was more likely to trapping electrons to generate OH than O_2_ (Eq. 10). Meanwhile, Fe^3+^ active sites were reduced by electrons to produce Fe^2+^ active sites which will activate H_2_O_2_ to produce regenerated Fe and new OH (Eqs. 11–12). Moreover, the generated Fe^3+^ reacted with OH^−^ to formed Fe^2+^ and OH (Eq. 13). Finally, TCH was degraded by the generated OH, ·O_2_^−^ and a small amount of h^+^ (Eq. 14) (Li J. et al., 2019). Therefore, the synergistic effect of photochemical and catalytic reaction exists in the system of Vis-light/ZnFe_2_O_4_/sepiolite/H_2_O_2_.

$$\text{catalyst}+{\text{h}}_{\text{ν}}\to \text{catalyst}\left({\text{e}}^{-}+{\text{h}}^{+}\right)$$ (6)

$${\text{h}}^{+}+{\text{H}}_{2}\text{O}\to \cdot \text{OH}+{\text{H}}^{+}$$ (7)

$${\text{h}}^{+}+{\text{OH}}^{-}\to \;\cdot \text{OH}$$ (8)

$${\text{e}}^{-}+{\text{O}}_{2}\to \;\cdot {\text{O}}_{2}^{-}$$ (9)

$${\text{e}}^{-}+{\text{H}}_{2}{\text{O}}_{2}\to \;\cdot \text{OH}+{\text{OH}}^{-}\;$$ (10)

$${\text{e}}^{-}+\;{\text{Fe}}^{3+}\to \;{\text{Fe}}^{2+}\;$$ (11)

$${\text{Fe}}^{2+}+{\text{H}}_{2}{\text{O}}_{2}\to {\text{Fe}}^{3+}+\cdot \text{OH}+{\text{OH}}^{-}$$ (12)

$${\text{Fe}}^{3+}+{\text{H}}_{2}{\text{O}}_{2}\to \;{\text{Fe}}^{2+}+\cdot \text{OOH}+{\text{H}}^{+}$$ (13)

$${\text{h}}^{+},\;\cdot \text{OH},\;\cdot {\text{O}}_{2}^{-}\;+\;\text{TCH}\to {\text{CO}}_{2}+{\text{H}}_{2}\text{O}$$ (14)

### Stability of Catalyst

**FIGURE 14:**
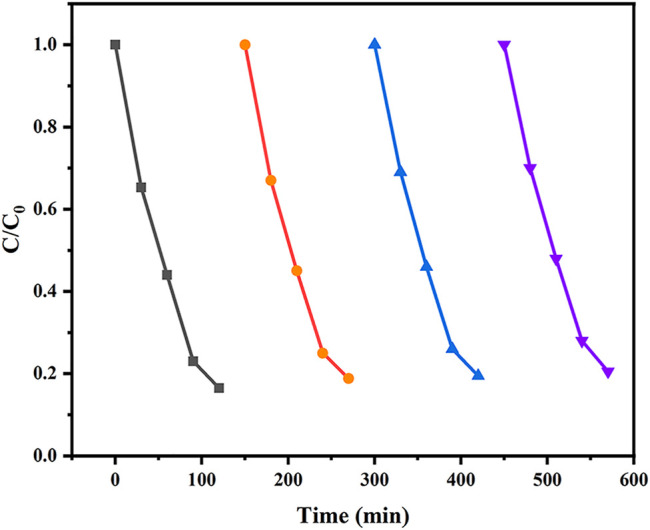
The reusability test results of ZF-Sep-11.

In order to explore the reusability and stability of the catalyst, four cycles of experiments were carried out. The degradation plots are shown in Figure 14, removal efficiency for the first cycle is 84.5%, second cycle is 81.2%, third cycle is 80.5%, and for the fourth cycle is 79.5%. It observed that there is no significant reduction in the removal efficiency. The above results show that the prepared catalyst has good recyclability and stability.

## Conclusion

In summary, we have successfully prepared the ZF/Sep composites *via* a co-precipitation method. The introduction of sepiolite nanofibers significantly improved the agglomeration of ZnFe_2_O_4_ and reduced the recombination rate of photogenerated electrons and holes, so all the ZF-Sep composites presented better removal efficiency for TCH than pure ZnFe_2_O_4_. The most suitable removal efficiency of TCH (20 mg/L) appeared at 1.0 g/L ZF-Sep-11 catalyst dosage, and 1 mM H_2_O_2_ for 3 h. Besides, 79.5% of TCH removal efficiency could be still retained after four cycles, and the catalyst had soft magnetic properties and could be easily recovered when a magnetic field was applied. Thus, ZF/Sep composite display a promising photocatalysis performance in treating wastewater contained antibiotics.

## Data Availability

The original contributions presented in the study are included in the article/supplementary material, further inquiries can be directed to the corresponding author.
